# GALNT2 suppresses malignant phenotypes through IGF-1 receptor and predicts favorable prognosis in neuroblastoma

**DOI:** 10.18632/oncotarget.2627

**Published:** 2014-10-24

**Authors:** Wan-Ling Ho, Chih-Hsing Chou, Yung-Ming Jeng, Meng-Yao Lu, Yung-Li Yang, Shiann-Tarng Jou, Dong-Tsamn Lin, Hsiu-Hao Chang, Kai-Hsin Lin, Wen-Ming Hsu, Min-Chuan Huang

**Affiliations:** ^1^ Department of Pediatrics, National Taiwan University Hospital and National Taiwan University College of Medicine, Taipei, Taiwan; ^2^ Department of Pathology, National Taiwan University Hospital and National Taiwan University College of Medicine, Taipei, Taiwan; ^3^ Department of Surgery, National Taiwan University Hospital and National Taiwan University College of Medicine, Taipei, Taiwan; ^4^ Graduate Institute of Clinical Medicine, National Taiwan University College of Medicine, Taipei, Taiwan; ^5^ Graduate Institute of Anatomy and Cell Biology, National Taiwan University College of Medicine, Taipei, Taiwan; ^6^ Department of Pediatrics, Shin Kong Wu Ho-Su Memorial Hospital, Taipei, Taiwan; ^7^ School of Medicine, Fu Jen Catholic University, New Taipei City, Taiwan; ^8^ Research Center for Developmental Biology and Regenerative Medicine, National Taiwan University, Taipei, Taiwan

**Keywords:** glycosyltransferase, insulin-like growth factor-1 receptor, N-Acetylgalactosaminyltransferase 2, neuroblastoma

## Abstract

Aberrant expression of the simple mucin-type carbohydrate antigens such as Tn antigen is associated with malignant transformation and cancer progression. *N*-acetylgalactosaminyltransferase 2 (GALNT2), one of the enzymes that mediate the initial step of mucin-type *O*-glycosylation, is responsible for forming Tn antigen. GALNT2 is expressed differentially in nervous tissues during mouse embryogenesis; however, the role of GALNT2 in neuroblastoma (NB) remains unclear. Here we showed that increased GALNT2 expression evaluated using immunohistochemistry in NB tumor tissues correlated well with the histological grade of differentiation as well as younger age at diagnosis, early clinical stage, primary tumor originated from the extra-adrenal site, favorable INPC histology, and *MYCN* non-amplification. Multivariate analysis showed that GALNT2 expression is an independent prognostic factor for better survival for NB patients. GALNT2 overexpression suppressed IGF-1-induced cell growth, migration, and invasion of NB cells, whereas GALNT2 knockdown enhanced these NB phenotypes. Mechanistic investigations demonstrated that GALNT2 overexpression modified *O*-glycans on IGF-1R, which suppressed IGF-1-triggered IGF-1R dimerization and subsequent downstream signaling events. Conversely, these properties were reversed by GALNT2 knockdown in NB cells. Our findings suggest that GALNT2 regulates malignant phenotypes of NB cells through the IGF-1R signaling pathway, suggesting a critical role for GALNT2 in the pathogenesis of NB.

## INTRODUCTION

Neuroblastoma (NB) is the most common extracranial solid tumor in childhood and the most common solid tumor of infancy, accounting for about 8–10% of childhood cancers. NB is an embryonal cancer of the peripheral sympathetic nervous system [1, 2]. This tumor exhibits extreme heterogeneity, from spontaneous differentiation or regression into ganglioneuroblastoma (GNB) or ganglioneuroma (GN) with a favorable prognosis to highly undifferentiated tumors with a rapid progression and very poor outcomes [3]. Approximately 60% of NB patients are clinically diagnosed as the stage 4 disease and have a very poor prognosis with a 5-year survival rate of no more than 30%. Although the overall prognosis of NB patients has improved remarkably with recent therapeutic advances, long-term survival of high-risk NB remains poor even with intensive multimodal therapy [1, 2, 4]. Identifying new prognostic factors is therefore important for understanding NB pathogenesis and developing tailored therapies that improve treatment outcomes for patients with unfavorable NB.

Glycosylation is one of the most important processes in posttranslational modification of proteins, and aberrant glycosylation affects many cellular properties, including cell proliferation, differentiation, transformation, migration, invasion, apoptosis, and immune responses [5]. There are two major types of protein glycosylation in mammalian cells, namely *N*-linked and *O*-linked. Mucin-type *O*-glycosylation is initiated by the transfer of *N*-acetylgalactosamine (GalNAc) to a serine (S) or threonine (T) residue, thereby forming the Tn antigen (GalNAcα-S/T) [6]. This reaction is catalyzed by a family of polypeptide GalNAc transferases (GALNTs) that consists of at least 20 members in humans, namely GALNT1 to 14 and GALNTL1 to L6 [7, 8]. Many of these GALNTs have been assigned biological functions, and aberrant expression of some GALNTs has been associated with human diseases. For example, the expression of GALNT3 is a potential diagnostic and prognostic marker for lung [9] and pancreatic [10] cancers. GALNT6 disrupts mammary acinar morphogenesis through *O*-glycosylation of fibronectin [11]. GALNT14 modulates death-receptor *O*-glycosylation in pancreatic carcinoma, non-small-cell lung carcinoma, and melanoma cells, and may therefore serve as a predictive biomarker for Apo2L/tumor necrosis factor-related apoptosis-inducing ligand-based cancer therapy [12].

Insulin-like growth factor (IGF) receptor is a promising therapeutic target as its overexpression is associated with the development and poor prognosis of various cancers. There are two IGF receptors, with IGF-1R and IGF-2R serving as positive and negative regulators of the IGF signaling pathway, respectively [13-15]. IGF-1R is a major determinant of the metastatic potential in NB [16]. In addition, IGF-1R expression enhanced by C-MYB and MYCN is associated with a highly malignant disease and poor patient prognosis [17-19]. Numerous phase I and phase II studies for pediatric cancers are currently underway with either IGF-1R inhibitors alone or combination regimen, however, the clinical efficacy is still unsatisfactory [20, 21]. To improve the efficacy of IGF-1R-targeted therapies, the molecular mechanisms by which IGF-1R regulates NB properties must be further characterized.

We previously demonstrated that GALNT2 dysregulation contributes to the malignant behaviors of hepatocellular carcinoma and oral squamous carcinoma cells by modifying *O*-glycosylation of the epidermal growth factor receptor [22, 23]. *GALNT2* transcripts are expressed differentially in nervous tissues during mouse embryogenesis [24]. The expression of GALNT2 also regulates migration and invasion of human glioma cells *in vitro* [25]. However, the role of GALNT2 in cell behaviors and clinical significance of NB remains unclear. Here we report a positive correlation between GALNT2 expression and the histological grade of differentiation in NB tissues. In addition, GALNT2 expression may predict better survival for patients with NB. Finally, GALNT2 modifies IGF-1R *O*-glycosylation and activity, thereby impacting the malignant phenotypes of NB cells both *in vitro* and *in vivo*.

## RESULTS

### GALNT2 expression and clinicopathologic and biologic factors of NB tumors

**Figure 1:**
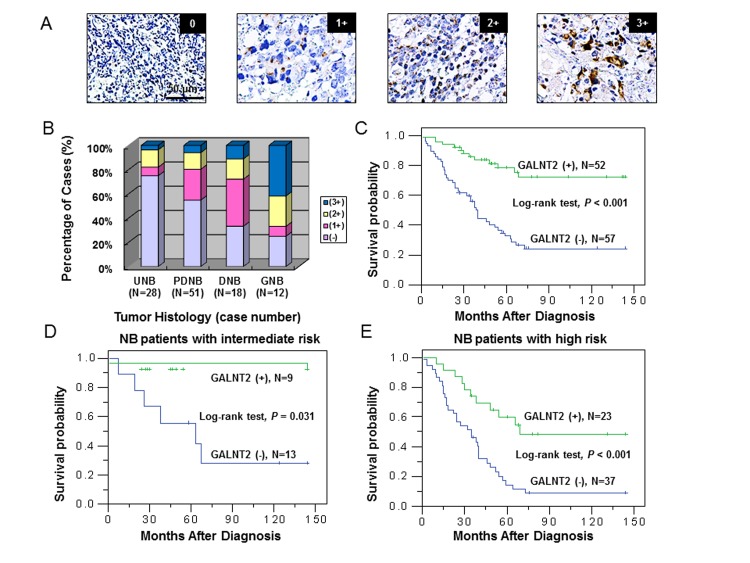
GALNT2 expression is correlated with tumor histology and survival probability of NB patients (A) Immunohistochemical images of NB tumors representing the four categories of GALNT2 expression (0 to 3+). Scale bar = 50 μm. Original magnification, 400×. (B) Percentage distribution of GALNT2 expression in tumors with UNB, PDNB, DNB, or GNB histology. (C) Kaplan-Meier survival analysis according to the expression of GALNT2 in 109 NB patients. *P* value was calculated using log-rank test. (D) Kaplan-Meier survival analysis according to the expression of GALNT2 in NB patients with intermediate risk. *P* value was calculated using log-rank test. (E) Kaplan-Meier survival analysis according to the expression of GALNT2 in NB patients with high risk. *P* value was calculated using log-rank test.

To investigate the clinical importance of GALNT2 and its correlation with clinicopathologic factors of NB, we examined GALNT2 expression in NB tumors using immunohistochemical staining. Positive GALNT2 staining in NB cells was observed specifically in the Golgi (Figure 1A) but not in Schwannian stromal cells. GALNT2 immunoreactivity is shown in Figure 1A. The immunoreactivity of GALNT2 in NB tumors was categorized into 4 groups: “0” (no expression); “1+” (weak expression, expression in ~10–35% of neuroblastic cells); “2+” (moderate expression, expression in ~35–70% of neuroblastic cells); and “3+” (strong expression, expression in >70% of neuroblastic cells). GALNT2 expression (1+, 2+, and 3+) was observed in most GNB (75%) and DNB tumors (67%) and was observed less frequently in PDNB (45%) and UNB (25%) tumors, indicating that the intensity and percentage of positive GALNT2 immunostaining correlated with the histological grade of differentiation (Figure 1B). For further analyses of the association between GALNT2 expression and other clinicopathologic and biologic factors, NB tumors were assigned to negative GALNT2 expression (“0” in immunoreactivity) and positive GALNT2 expression (“1+”, “2+”, or “3+” in immunoreactivity). The immunohistochemical staining revealed GALNT2-positive expression (1+ to 3+) in 47.7% (52/109) of NB tumors. We found that in addition to histological grade of differentiation, positive GALNT2 expression significantly correlated with younger age at diagnosis (≤ 1.5 year; *P* = 0.013, χ^2^ test), early clinical stage (stage 1, 2, and 4S; *P* = 0.016, χ^2^ test), primary tumor originated from the extra-adrenal site (*P* = 0.019, χ^2^ test), favorable INPC histology (*P* = 0.001, χ^2^ test) and *MYCN* non-amplification (*P* = 0.025, χ^2^ test) (Table 1).

**Table 1:** GALNT2 expression and the clinicopathologic and biologic characteristics of neuroblastoma

Variable	Cases	Positive GALNT2expression (%)	*P* value*
Age at diagnosis			
≤ 1.5 year	35	23 (65.7)	0.013
> 1.5 year	74	29 (39.2)	
Sex			
Male	65	29 (44.6)	0.432
Female	44	23 (52.2)	
Clinical stage			
1, 2, and 4S	32	21 (65.6)	0.016
3 and 4	77	31 (40.3)	
Primary tumor site			
Adrenal	67	26 (38.8)	0.019
Extra-adrenal	42	26 (61.9)	
INPC histology			
Unfavorable	50	15 (30.0)	0.001
Favorable	59	37 (62.7)	
*MYCN*			
Amplified	25	7 (28.0)	0.025
Non-Amplified	84	45 (53.6)	

Abbreviation: INPC, International Neuroblastoma Pathology Classification.

* χ2 test.

### GALNT2 expression and patient survival analysis

Kaplan-Meier analysis showed that patients with GALNT2-positive tumors had a higher predictive 5-year survival rate than those with GALNT2-negative tumors (*P* < 0.001, log-rank test; Figure 1C). Furthermore, univariate analysis showed that in addition to the absence of GALNT2 expression, older age at diagnosis (>1.5 year), advanced clinical stage (stage 3 and 4), *MYCN* amplification, and unfavorable INPC histology strongly correlated with poor survival (Table 2). Multivariate analysis revealed that advanced clinical stage, *MYCN* amplification, unfavorable INPC histology, and negative GALNT2 expression remained independent prognostic factors for poor survival (Table 2). To further evaluate the significance of GALNT2 expression in prognostic discrimination, the impact of GALNT2 expression on survival rate was analyzed according to the COG risk grouping. Except for low-risk patients who had very good prognoses, positive GALNT2 expression predicted higher survival probability for patients with either intermediate- (*P* = 0.031, log-rank test; Figure 1D) or high-risk group (*P* < 0.001, log-rank test; Figure 1E). These results suggested that GALNT2 expression is an independent prognostic factor for survival in patients with NB and may provide information that complements the COG risk classification.

**Table 2:** Clinicopathologic and biologic factors affecting survival rate

	Univariate analysis			Multivariate analysis		
Variable	RR	95% CI	*P* value	RR	95% CI	*P* value
Age at diagnosis > 1.5 year versus ≤ 1.5 year	4.098	1.938 – 8.662	0.001	1.242	0.459 – 3.357	0.670
Clinical stage Advanced (3 & 4) versus early (1, 2 and 4S)	13.328	4.146 – 42.842	< 0.001	5.113	1.314 – 19.894	0.019
*MYCN* Amplified versus non-amplified	3.457	2.048 – 5.834	< 0.001	2.034	1.135 – 3.645	0.017
GALNT2 expression Negative versus positive	4.320	2.265 – 8.239	< 0.001	2.495	1.248 – 4.987	0.010
INPC histology Unfavorable versus favorable	3.720	2.115 – 6.543	< 0.001	2.220	1.193 – 4.133	0.012
Primary tumor site Adrenal versus non-adrenal	1.299	0.764 – 2.211	0.334	ND	ND	ND

Abbreviations: INPC, International Neuroblastoma Pathology Classification; RR, risk ratio; 95% CI, 95% confidence interval; ND, not done.

### Stable transfection of NB cells with GALNT2

Three NB cell lines (SH-SY5Y, SK-N-AS, and SK-N-DZ) were used for various experiments in this study, so we examined the general glycophenotypes of SH-SY5Y and SK-N-DZ cells by flow cytometry with the following lectins: VVA-FITC, which is specific for Tn antigen, PNA-FITC, which preferentially binds to T antigen, *Maackia amurensis* lectin (MAL)-FITC, which is specific for *N-*acetylneuraminic acid (α-2,3) galactose structure, and *Sambucus nigra* lectin (SNA)-FITC, which is mainly specific for *N-*acetylneuraminic acid (α-2,6) galactose structure. After pretreatment with neuraminidase, more glycoproteins on the cell surface could be pulled down by VVA and PNA (Supplementary Figure S1A and S1B, upper panels). Moreover, glycoproteins on the cell surface could be pulled down by MAL and SNA (Supplementary Figure S1A and S1B, lower panels). These results suggested that Tn, T, sialyl-Tn (sTn), sialyl-T (sT), and α(2,3)- and α(2,6)-linked sialic acid structures were expressed in parental SH-SY5Y and SK-N-DZ cells.

**Figure 2:**
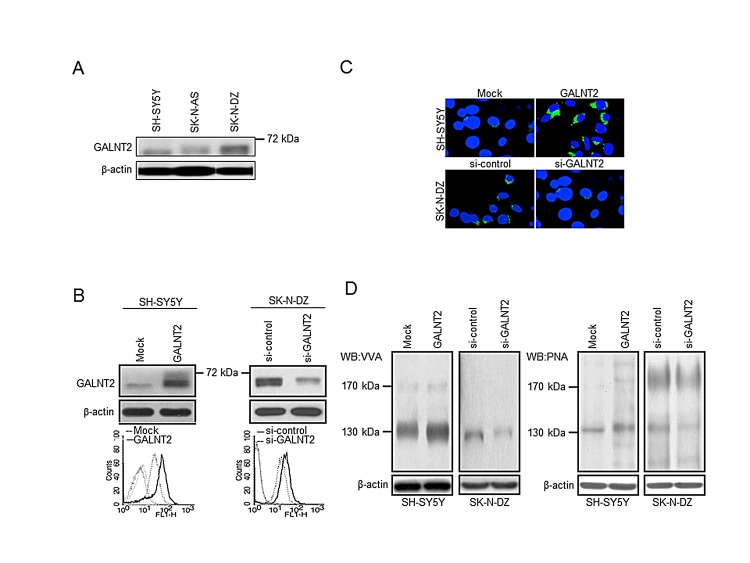
Stable transfection of NB cells with GALNT2 (A) Expression of GALNT2 in NB cell lines, as determined by Western blotting. (B) Overexpression of GALNT2 in SH-SY5Y cells and knockdown of GALNT2 in SK-N-DZ cells. In upper panels, SH-SY5Y cells were transfected with pcDNA3.1B/myc-His control plasmid (Mock) or *GALNT2/*pcDNA3.1B plasmid (GALNT2); SK-N-DZ cells were transfected with control siRNA (si-control) or GALNT2 siRNA (si-GALNT2). The overexpression or knockdown of GALNT2 was confirmed by Western blotting. β-actin is an internal control. Lower panels show results from flow cytometry using VVA-FITC to detect GalNAc-O-Ser/Thr (Tn antigen). (C) Immunofluorescence staining was performed to observe the subcellular topology in GALNT2-overexpressing SH-SY5Y cells, GALNT2-knockdown SK-N-DZ cells, and their controls. The localization of GALNT2 was enhanced in the Golgi apparatus of GALNT2-overexpressing SH-SY5Y transfectants (green) compared with mock transfectants. By contrast, GALNT2 localization in the Golgi apparatus was suppressed by GALNT2 knockdown in SK-N-DZ cells. (D) Changes in carbohydrates on cellular proteins were detected using VVA, which is specific for GalNAc-O-Ser/Thr (Tn antigen), or PNA, which is specific for galactosyl (β-1,3)-N-acetylgalactosamine structure (T antigen).

To investigate the role of GALNT2 in NB, we examined GALNT2 expression in three NB cell lines by Western blotting. The results demonstrated that the expression level of GALNT2 was lower in SH-SY5Y and SK-N-AS cell lines and higher in SK-N-DZ cell line (Figure 2A). We further overexpressed GALNT2 in SH-SY5Y and SK-N-AS cells and knocked down GALNT2 in SK-N-DZ cells. The overexpression and knockdown of GALNT2 were confirmed by Western blotting (Figure 2B and Supplementary Figure S2A, upper panels) and immunofluorescence microscopy (Figure 2C). To confirm that GALNT2 has GalNAc transferase activity, two lectins were used: VVA, which preferentially binds to *N*-acetylgalactosamine attached to a serine or threonine residue (Tn antigen) and PNA, which preferentially binds to Galβ-1,3GalNAc (T antigen). We found that GALNT2 overexpression increased GalNAc expression on cellular proteins in SH-SY5Y cells. In contrast, GALNT2 knockdown decreased GalNAc expression on cellular proteins in SK-N-DZ cells (Figure 2D). We also performed flow cytometry for detecting surface expression of GalNAc structure in NB cells using VVA-FITC. The results showed that GALNT2 overexpression increased the amount of GalNAc on the surface of SH-SY5Y and SK-N-AS cells, whereas GALNT2 knockdown decreased the amount on SK-N-DZ cells compared with controls (Figure 2B and Supplementary Figure S2A, lower panels). We also noted that SH-SY5Y and SK-N-AS transfectants expressing a higher level of GALNT2 showed a higher amount of Tn antigen on the surface of NB cells (Supplementary Figure S1C and S1D). Furthermore, we found that GALNT2 mainly enhanced GalNAc expression on cellular glycoproteins located at 130 kDa (Figure 2D). There are several cellular proteins with a molecular weight of ~130 kDa, and IGF-1R has been reported to play an essential role in the pathogenesis and cell behaviors in NB [19, 26]. We therefore postulated that IGF-1R may be a critical acceptor substrate for GALNT2 in NB cells [27, 28].

### GALNT2 suppresses malignant phenotypes of NB cells

**Figure 3:**
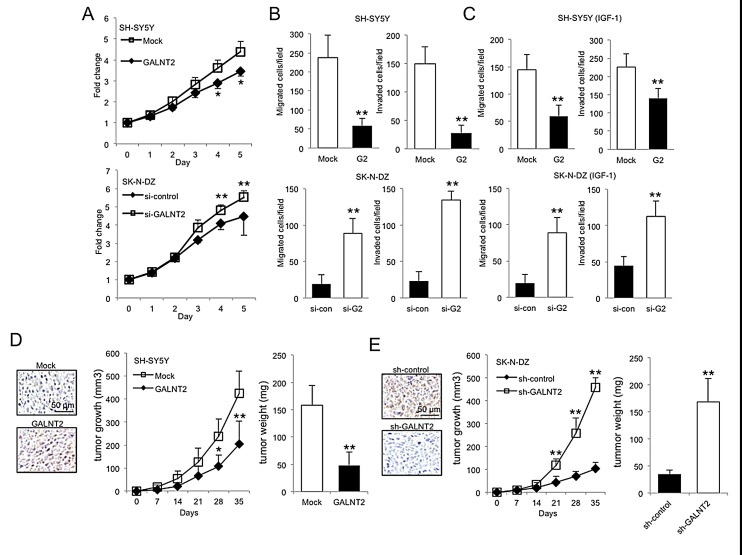
Effects of GALNT2 on malignant phenotypes of NB cells (A) MTT assays indicated that GALNT2 overexpression significantly suppressed SH-SY5Y cell growth (top panel), whereas GALNT2 knockdown in SK-N-DZ cells enhanced cell growth compared with controls (bottom panel). Cells were cultured in DMEM containing 10% FBS, and MTT reagent was added at the indicated times. The results were standardized by setting the value at day 0 to 1.0. Data are presented as mean ± SD from three independent experiments. Error bar = SD. *P < 0.05, **P < 0.01. (B–C) GALNT2 overexpression in SH-SY5Y cells (G2) significantly inhibited FBS- and IGF-1-induced migration and invasion compared with controls (Mock) (upper panels). GALNT2 knockdown in SK-N-DZ cells (si-G2) enhanced migration and invasion induced by FBS or IGF-1 compared with controls (si-con) (lower panels). Migration and invasion were analyzed by transwell migration and Matrigel invasion assays, respectively. Cells were seeded in serum-free DMEM and the chemoattractant in the lower chamber was 10% FBS or 50 ng/mL IGF-1. Data are presented as mean ± SD from three independent experiments. Error bar = SD. **P < 0.01. (D–E) GALNT2 suppressed tumor growth in mice. SH-SY5Y (D) and SK-N-DZ (E) transfectants were subcutaneously injected to mice. After implantation, tumor sizes were measured twice a week. At day 35, tumors were excised, weighed, and subjected to immunohistochemical staining using the anti-GALNT2 antibody (color images). Scale bar = 50 μm. Original magnification, 400×. Data represent the mean ± SD; *n* = 4 for each group. *P < 0.05, **P < 0.01.

To investigate effects of GALNT2 on malignant phenotypes of NB cells, cell growth, migration, and invasion were analyzed. MTT assays revealed that GALNT2 overexpression significantly suppressed SH-SY5Y and SK-N-AS cell growth (Figure 3A, top panel and Supplementary Figure S2B), whereas GALNT2 knockdown significantly enhanced SK-N-DZ cell growth (Figure 3A, bottom panel). In addition, GALNT2 overexpression in SH-SY5Y and SK-N-AS cells significantly inhibited FBS- and IGF-1-induced migration and invasion as revealed by transwell migration and Matrigel invasion assays, respectively (Figure 3B and 3C, upper panels and Supplementary Figure S2C). Conversely, GALNT2 knockdown significantly enhanced FBS- and IGF-1-induced migration and invasion in SK-N-DZ cells (Figure 3B and 3C, lower panels and Supplementary Figure S3). However, we did not observe significant changes in migration and invasion in serum-free conditions (data not shown). These results suggested that GALNT2 suppresses malignant phenotypes of NB cells through the IGF-1-mediated pathway.

### GALNT2 inhibits tumor growth *in vivo*

To examine the effect of GALNT2 on tumor growth *in vivo*, mice were injected with GALNT2-transfected SH-SY5Y or SK-N-AS cells, shGALNT2-transfected SK-N-DZ cells as well as control cells. We found that GALNT2 overexpression significantly decreased the size and weight of tumors (Figure 3D and Supplementary Figure S2D), and GALTN2 knockdown resulted in larger tumors (Figure 3E). These results suggested that GALNT2 suppresses NB tumor growth *in vivo*.

### GALNT2 modifies glycosylation and activity of IGF-1R in NB cells

**Figure 4:**
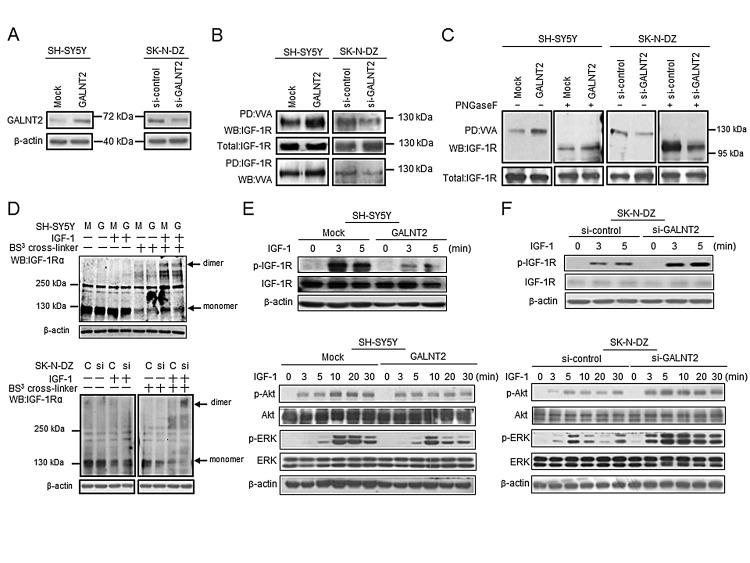
GALNT2 modifies IGF-1R glycosylation and activity and modulates IGF-1R phosphorylation and signaling in NB cells (A) Overexpression of GALNT2 in SH-SY5Y cells and knockdown of GALNT2 in SK-N-DZ cells. The overexpression or knockdown of GALNT2 was confirmed by Western blotting. β-actin is an internal control. (B) GALNT2 overexpression increased Tn expression on IGF-1R in SH-SY5Y cells. IGF-1R knockdown in SK-N-DZ cells decreased Tn expression on IGF-1R. Cell lysates were pulled down with VVA and then immunoblotted with anti-IGF-1R antibody (top set). Cell lysates were immunoprecipitated with anti-IGF-1R antibody, and then immunoblotted with VVA (bottom set). (C) IGF-1R carries *O*-glycans which can be modulated by GALNT2. GALTN2 overexpression increased VVA binding to IGF-1R in SH-SY5Y cells treated (+) or not treated (–) with PNGaseF (left two sets). GALNT2 knockdown decreased VVA binding to IGF-1R in SK-N-DZ cells treated (+) or not treated (–) with PNGaseF (right two sets). Cell lysates pretreated with PNGaseF were pulled down with VVA and then immunoblotted with anti-IGF-1R antibody. Fully or partially glycosylated IGF-1R exhibits a molecular mass of ~130 kDa or ~95–100 kDa, respectively. SH-SY5Y cells were transfected with pcDNA3.1B/myc-His control plasmid (Mock) or *GALNT2*/pcDNA3.1B plasmid (GALNT2); SK-N-DZ cells were transfected with control siRNA (si-control) or GALNT2 siRNA (si-GALNT2). (D) GALNT2 overexpression reduced IGF-1Rα dimerization triggered by IGF-1 in SH-SY5Y cells. Cells were treated (+) or not treated (–) with IGF-1. The BS^3^ cross-linker was used to detect receptor dimerization. M, mock control; G, GALNT2 overexpression (upper panel). GALNT2 knockdown enhanced IGF-1-induced IGF-1Rα dimerization in SK-N-DZ cells. C, control; si, GALNT2 knockdown (lower panel). (E) GALNT2 suppressed IGF-1-induced phosphorylation of IGF-1R and its downstream signaling molecules in SH-SY5Y cells. (F) GALNT2 knockdown enhanced IGF-1-induced phosphorylation of IGF-1R and its downstream signaling molecules in SK-N-DZ cells. In (D) and (E), cells were starved for 16 h and then treated with IGF-1 for the indicated amount of time. Western blotting was carried out using antibodies specific to IGF-1R, Akt, and ERK and their phosphorylated forms. β-actin was used as the loading control.

Because we found that IGF-1R may be a critical acceptor substrate for GALNT2 in NB cells, we first examined the expression levels of endogenous IGF-1R in SH-SY5Y and SK-N-DZ cells by Western blotting. The result showed that the IGF-1R expression level was similar in these two cell lines (Supplementary Figure S4A). We further tested whether GALNT2 regulates IGF-1R glycosylation and activity. We found that GALNT2 overexpression increased the amount of IGF-1R pulled down by VVA in SH-SY5Y cells; in contrast, GALNT2 knockdown decreased the amount of IGF-1R pulled down by VVA in SK-N-DZ cells (Figure 4A and 4B). To confirm the selectivity of the VVA lectin, GalNAc (100 μM) was used as a competitor for VVA-lectin pull-down assays and flow cytometry. The result showed that GalNAc effectively blocked VVA binding to Tn-antigen structures on IGF-1R in SH-SY5Y cells (Supplementary Figure S4B and S4C). To further demonstrate that *O*-glycans are present on IGF-1R and can be modulated by GALTN2, PNGaseF was used to remove *N*-glycans before the lectin pull-down and immunoblotting. We found that VVA still bound to IGF-1R after removal of *N*-glycans by PNGaseF (Figure 4C). Moreover, VVA binding to IGF-1R was even increased in SK-N-DZ cells. These results suggested that IGF-1R carries *O*-glycans which can be modulated by GALNT2.

To investigate the effects of GALNT2-mediated glycosylation on IGF-1R, we analyzed whether GALNT2 expression affects IGF-1-induced dimerization of IGF-1R by using the BS^3^ cross-linker. We found that IGF-1-induced IGF-1Rα dimerization was reduced in GALNT2-overexpressing SH-SY5Y cells (Figure 4D, upper panel), whereas GALNT2 overexpression did not affect IGF-1-induced IGF-1Rβ dimerization (data not shown). In SK-N-DZ cells, GALNT2 knockdown significantly enhanced IGF-1-induced IGF-1Rα dimerization (Figure 4D, lower panel) but did not affect IGF-1-induced IGF-1Rβ dimerization (data not shown). These results suggested that GALNT2 decorates *O*-glycans on IGF-1R and, which in turn, suppresses IGF-1-triggered dimerization of IGF-1R.

### GALNT2 modulates IGF-1R phosphorylation and its downstream signaling in NB cells

Because GALNT2 expression suppresses IGF-1-triggered dimerization of IGF-1R, we next examined the effect of GALNT2 on IGF-1R phosphorylation induced by IGF-1. Our data showed that GALNT2 overexpression inhibited IGF-1-induced phosphorylation of IGF-1R in SH-SY5Y cells. Furthermore, phosphorylation of IGF-1R downstream signaling molecules, including p-Akt and p-ERK, was also suppressed in SH-SY5Y cells (Figure 4E). In contrast, GALNT2 knockdown in SK-N-DZ cells enhanced IGF-1-induced phosphorylation of IGF-1R, Akt and ERK (Figure 4F). Notably, IGF-1R inhibitor AG1024 significantly inhibited IGF-1-triggered cell growth (Supplementary Figure S5A), migration (Supplementary Figure S5B) and invasion (Supplementary Figure S5C) in GALNT2-knockdown SK-N-DZ cells. These results suggested that GALNT2 expression downregulates IGF-1-triggered phosphorylation of IGF-1R, thereby suppressing signaling through the IGF-1R pathway.

## DISCUSSION

Accumulating evidence has shown that aberrant expression of glycosyltransferases confers the altered *O*-glycans structures such as Tn, sTn, T, and sT antigens in many types of cancer cells [29]. Short *O*-glycans have also been detected in human NB [30], but the role that short *O*-glycans play in NB pathogenesis and clinical significance of NB remains largely unknown. *GALNT2* transcripts are expressed differentially in nervous tissues during mouse embryogenesis [24]. The expression of GALNT2 also regulates migration and invasion of human glioma cells *in vitro* [25]. We therefore investigated the expression and roles played by GALNT2 and short *O*-glycans in NB. By immunohistochemical staining, we found that the intensity and percentage of positive GALNT2 staining significantly correlated with the histological grade of differentiation. In addition, positive GALNT2 expression significantly correlated with younger age at diagnosis, early clinical stage, primary tumor originated from the extra-adrenal site, favorable INPC histology and *MYCN* non-amplification. Survival analysis revealed that GALNT2 expression was an independent prognostic factor for better survival for NB patients. The COG risk grouping is widely adapted for prognosis discrimination and treatment allocation of NB patients [3]. However, NB patients in either intermediate- or high-risk group present with prognostic heterogeneity. Our results revealed that positive GALNT2 expression predicted a favorable prognosis in patients with either intermediate- or high-risk group. Therefore, assessing GALNT2 expression in NB tumors may provide information that complements the COG risk classification and helps physicians to subgroup NB patients as homogenously as possible in terms of biology and outcome.

In the present study, we found that GALNT2, a GalNAc transferase, can regulate Tn expression in NB cells (Figure 2) and suppress the malignant properties of these cells via the IGF-1-mediated pathway (Figure 3). Moreover, IGF-1R may be a critical acceptor substrate for GALNT2 in NB cells (Figure 2) because GALNT2 expression enhances the GalNAc expression on IGF-1R (Figure 4B). More importantly, GALNT2 enhanced VVA binding to IGF-1R even after removal of *N*-glycans by PNGaseF, indicating that IGF-1R carries *O*-glycans which can be modulated by GALNT2 (Figure 4C). This is an interesting result because it has long been thought that IGF-1R is modified with only *N*-glycans [31]. A recent study reported that without modification of *N*-glycosylation of the IGF-1R at N913, IGF-1R/insulin receptor (IR) heterodimeric receptors would fail to localize to the plasma membrane, thus preventing receptor-ligand binding and decreasing the efficacy of anti-IGF-1R antibody-based cancer therapies. This highlights the impact of glycosylation of IGF-1R on cancer behaviors and therapies [32]. It is therefore important to determine the IGF-1R residues that are *O*-glycosylated and to assess the biological and clinical significance of these glycosylation events for patients with NB.

IGF-1Rs exist as either homodimers or heterodimers with IRs prior to ligand binding. Either variation can participate in tumorigenesis [13]. Ligand binding to IGF-1R results in receptor dimerization, autophophorylation of the intracellular domain, kinase activation, and phosphorylation of cellular substrates leading to gene activation and ultimately proliferation or differentiation of cells through two primary cascades, the MAP kinase and PI3 kinase pathways [33]. Our data showed that GALNT2 suppressed IGF-1-induced IGF-1R dimerization, suggesting that conformational changes in *O*-glycans on IGF-1R regulate IGF-1R dimerization and its downstream signaling related to cell growth, migration, and invasion.

Numerous studies have suggested that transformed cells express higher levels of IGF-1R than normal cells. In pediatric tumors such as Wilms' tumor, rhabdomyosarcoma, and NB, IGF-1R overexpression presumably increases the cellular responsiveness to the IGFs in terms of proliferation and inhibition of apoptosis [34]. IGF-1R activation and subsequent activation of the PI3 kinase and MAP kinase pathways induce the extension of lamellipodia in NB cell lines, which is related to the spreading ability [35]. In addition, NB cell lines highly expressing IGF-1R were much more likely to develop osteolytic lesions when injected into mouse tibia compared to the same cells without IGF-1R, suggesting that IGF-1R may play an important role in NB metastasis [16]. In addition, IGF-1R expression can be enhanced by C-MYB and MYCN and is associated with a highly malignant disease and poor prognosis [17-19]. Here we found that GALNT2 strongly correlated with favorable prognostic factors of NB, such as histological grade of differentiation, younger age at diagnosis, early clinical stage, primary tumor originated from the extra-adrenal site, favorable INPC histology and *MYCN* non-amplification. We also observed that GALNT2 inhibited malignant phenotypes of NB cells through the IGF-1R signaling pathway. Although the molecular mechanisms underlying the correlations between GALNT2 and individual factors need to be clarified, these findings do suggest that GALNT2 may be a promising therapeutic target for NB treatment.

Here we showed that GALNT2 modulated FBS-triggered cell migration and invasion in NB cells. It is therefore possible that GALNT2 exerts its effects through other receptors in addition to IGF-1R. For the past two decades, IGF-1R inhibitors have been developed for anticancer therapies in a variety of pediatric and adult cancers. However, initial clinical studies of IGF-1R antagonists as a single agent have not yielded satisfactory results, suggesting that multiple pathways inherent in NB may explain the varying treatment sensitivity and drug resistance in NB patients. Clinical studies are currently underway combining IGF-1R antagonists with other agents for synergy [13]. Results from our current study may provide an alternative approach for cancer therapy by means of modulating cancer-specific glycosylation.

In conclusion, we show that expression of GALNT2 serves as an independent prognostic factor that predicts favorable outcomes for patients with NB. GALNT2 can modify IGF-1R *O*-glycosylation and interfere with IGF-1-triggered dimerization and activity of IGF-1R, thereby regulating downstream signaling events related to malignant properties of NB cells *in vitro* and tumor growth *in vivo*. However, because NB cells may have other acceptor substrates of GALNT2, further investigation to identify these other substrates and associated signaling molecules is warranted for comprehensive understanding of the effects of GALNT2 in NB. This study also offers novel insights into the role of short *O*-glycans and GALNT2 in the pathogenesis of NB.

## MATERIALS AND METHODS

### Patients and tissue samples

From January 1, 2001 to June 30, 2012, there were 120 NB patients that received treatment at the National Taiwan University Hospital. Of these patients, 109 were enrolled in this study because they had a complete follow-up and sufficient tumor tissue from biopsies and/or surgical resections. The use of human tissues for this study was approved by the National Taiwan University Hospital Ethics Committee, and written consent was obtained from patients before sample collection. The median age at diagnosis was 2.3 years (range 0-11.3 years). Male patients were slightly predominant, with a male/female ratio of 65:44. Most tumors (67 cases) originated primarily from the adrenal gland. Tumor histology was categorized into four types according to the criteria of the International Neuroblastoma Pathology Classification (INPC) [36, 37] including: undifferentiated NB (UNB), poorly differentiated NB (PDNB), differentiating NB (DNB), and ganglioneuroblastoma (GNB), intermixed. The GNB, nodular subtype, was classified into UNB, PDNB, or DNB according to the morphologic features of the NB nodules because the tumor behavior of this subtype depends mainly on the NB nodules. The GN, maturing or mature subtype, is a benign lesion and was not included in this study. For prognostic analysis, GNB, intermixed was classified as a favorable histologic type, whereas UNB, PDNB, and DNB were classified as either favorable or unfavorable according to the mitosis-karyorrhexis index and the patient age at diagnosis based on the criteria of the INPC [36, 37]. Tumor staging was based on the International NB Staging System [38]. *MYCN* status was evaluated using chromogenic *in situ* hybridization analysis of formalin-fixed paraffin-embedded tissues or fresh single tumor cells [39]. Based on risk classifications of the Children's Oncology Group (COG), patients were classified into low-, intermediate-, and high-risk groups and were treated with surgery only or a combination of multiple modalities including chemotherapy, radiotherapy, and/or autologous bone marrow transplantation [40]. The median follow-up after diagnosis was 48 months (range of 1–144 months), and the overall predictive 5-year survival rate for this cohort was 53.6 %.

### Immunohistochemistry

Paraffin-embedded tissue sections were deparaffinized in xylene and rehydrated in a series of graded alcohols. After incubation of 3% H_2_O_2_ in PBS with 0.1% Triton X-100 at room temperature for 30 minutes, the sections were blocked with 5% bovine serum albumin (BSA) in PBS for 1 hour. The sections were incubated with a rabbit polyclonal anti-GALNT2 antibody (Sigma-Aldrich) at 1:200 in 1% BSA/PBS at 37ºC for 16 hours. After rinsing twice with PBS, the Super Sensitive Link-Label immunohistochemistry Detection System (BioGenex) was applied to tissue sections. Specific immunostaining was visualized with 3,3-diaminobenzidine liquid substrate system (Sigma-Aldrich). All sections were counterstained with hematoxylin and mounted with UltraKitt (J.T. Baker). For negative controls the primary antibodies were replaced with a control non-immune IgG at the same concentration. The immunoreactivity of GALNT2 was examined in a blinded manner by two independent investigators (Dr. Hsu WM and Dr. Jeng YM) without knowledge of clinical background of the patients. GALNT2 expression levels in NB tumors examined by immunohistochemical staining correlated well with those examined by Western blotting, as described in our previous study [22].

### Cell lines and cell culture

NB cell lines SH-SY5Y and SK-N-DZ were kindly provided by Dr. Yung-Feng Liao (Academia Sinica, Taiwan). SK-N-AS cells were kindly provided by Dr. Chung-Yi Hu (National Taiwan University, Taiwan). All cell lines were authenticated by Dr. Chung-Yi Hu based on morphology, antigen expression, growth, DNA profiles, and cytogenetics. Cells were maintained in Dulbecco's modified Eagle's medium (DMEM; Invitrogen, Life Technologies Inc.) containing 10% FBS (Invitrogen, Life Technologies Inc.), 100 IU/mL penicillin, and 100 μg/mL streptomycin (Invitrogen, Life Technologies Inc.) in a humidified tissue culture incubator at 37ºC and 5% CO_2_ atmosphere. All cell culture experiments were conducted with cells at less than 30 passages after receipt. Cells were tested to be mycoplasma free prior to experiments.

### Transfection

SH-SY5Y and SK-N-AS cells were transfected with *GALNT2*/pcDNA3.1B (GALNT2) or pcDNA3.1B/myc-His (Vector; Invitrogen, Life Technologies Inc.) using Lipofectamine 2000 (Invitrogen, Life Technologies Inc.) according to the manufacturer's protocol. Transfected cells were selected with 400 μg/mL of G418 for 14 days and pooled for further studies. The plasmids were constructed as previously described [22].

### Knockdown of GALNT2 expression

Specific duplex si-RNA against GALNT2 and a non-targeting control si-RNA (Invitrogen, Life Technologies Inc.) were obtained for further experiments. SK-N-DZ cells were transfected for 48 hours with 20 nmol/L si-RNA using Lipofectamine RNAiMAX (Invitrogen, Life Technologies Inc.). For stable knockdown of GALNT2, SK-N-DZ cells were infected with sh*GALNT2*/pLKO.1-puro (shGALNT2) or shCtrl/pLKO.1-puro (shCtrl; RNAi Core, Academia Sinica) using lentivirus-based infection system. After selected with 1 μg/mL puromycin (Millipore) for 2 weeks, cells were injected subcutaneously into mice for *in vivo* cell growth observation.

### Immunofluorescent staining

SH-SY5Y and SK-N-DZ cells were grown on 10-mm cover glasses and treated with plasmids or siRNA as described above. Cells were fixed with 4% paraformaldehyde for 15 minutes and permeabilized with 0.1% Triton X-100 in PBS for 10 minutes. After fixation, cells were blocked with 5% BSA in PBS for 1 hour at room temperature and incubated with the rabbit anti-GALNT2 antibody (1:100; Sigma-Aldrich) at 4ºC for 16 hours. Then cells were washed with PBS twice and incubated with anti-rabbit IgG-FITC antibody at 1:250 for 1 hour at room temperature. The images were captured by Zeiss microscope system.

### MTT assay

Cells were seeded in 96-well plates, and each well contained 2 × 10^3^ cells in 100 μl complete DMEM. We then added 10 μl of 5 mg/ml 3-(4,5-dimethyl-2-thiazolyl)-2,5-diphenyl-2H-tetrazolium bromide solution (MTT; Sigma-Aldrich) to each well for the indicated times. Cells were incubated with MTT solution at 37ºC for 4 hours. To dissolve the MTT formazan crystals, 100 μl 10% SDS in 0.01N HCl was added. The colorimetrical intensity was measured at the dual wavelengths of 550 and 630 nm using a spectrophotometer.

### Xenograft tumor growth in nude mice

For *in vivo* xenograft tumor growth analysis, 5×10^6^ of SH-SY5Y, SK-N-AS, and SK-N-DZ transfectants (*n* = 4 mice for each group) were subcutaneously injected into 6-week-old female nude mice (National Laboratory Animal Center, Taiwan). Day 35 after injection, the mice were sacrificed and tumors were excised for further analyses. Animal experiments were reviewed and approved by the Institutional Animal Care and Use Committee IACUC) of College of Medicine, National Taiwan University. Each tumor was weighted and sized and then lysed for immunohistochemistry using the anti-GALNT2 antibody (Sigma-Aldrich).

### Transwell migration and Matrigel invasion assay

Transwell migration assays were performed using 6.5-mm polycarbonate transwell filters with 8-μm pores (Corning Costar Corp.). Approximately 5×10^4^ SH-SY5Y, SK-N-AS, or SK-N-DZ cells in 300 μl serum-free DMEM were seeded to the upper surface of the transwell chamber, whereas 700 μl 10% FBS in complete DMEM was loaded into the lower chamber of 24-well plates. BioCoat Matrigel invasion chambers (BD Pharmingen) were used for the cell invasion assay. Cells were allowed to migrate toward the transwell chamber or invade the Matrigel for 24 hours. For IGF-1-induced malignant phenotypes of NB cells, 50 ng/ml IGF-1 was added to the lower chamber to trigger cell migration and invasion. The migrated and invaded cells were fixed with 100% methanol and stained with 0.5% (w/v) crystal violet (Sigma-Aldrich). The numbers of migrated and invaded cells per field were counted in at least three independent experiments (mean ± SD).

### Flow cytometry

SH-SY5Y and SK-N-AS cells were transfected with *GALNT2*/pcDNA3.1B or pcDNA3.1B/myc-His for 24 hours; SK-N-DZ cells were transfected with si-control or si-GALNT2 for 48 hours. Cells (1×10^5^) were resuspended in 100 μl PBS with 0.5% BSA, pretreated with neuraminidase at 37ºC for 1 hour, and then incubated with *Vicia villosa* agglutinin (VVA, Vector Laboratories)-FITC at 1:100 in PBS with 0.5% BSA on ice for 30 minutes. After cells were washed twice, the fluorescence intensity of 1×10^4^ cells per sample was analyzed using flow cytometry (FACS Calibur; BD Pharmingen). Samples without primary antibody served as negative controls for each cell

### Western blot analysis and lectin pull-down assay

A polyclonal antibody specific for GALNT2 (Sigma-Aldrich) was used to detect GALNT2. For detection of IGF-1 downstream signaling, antibodies against IGF-1Rα (Santa Cruz Biotechnology, Inc.); IGF-1Rβ, p-IGF-1Rβ, p-Akt, p-ERK, total ERK (Cell Signaling Technology); Akt123 (GeneTex Inc.); and β-actin (Sigma-Aldrich) were used. To analyze modifications to cell surface glycoproteins, lectin pull-down assays were used. Total cell lysates (300 μg) were pretreated with neuraminidase at 37ºC for 1 hour, then biotinylated-VVA or -peanut agglutinin (PNA) agarose beads (Vector Laboratories) were added and incubated at 4ºC overnight. Pulled-down proteins were subjected to Western blot analysis. Protein G sepharose beads (GE Heathcare) were used for immunoprecipitation. Bands were visualized by incubation with horseradish peroxidase (HRP)-conjugated streptavidin, HRP-conjugated secondary antibodies (Jackson ImmunoResearch Laboratories) and enhanced chemiluminescence reagents (Perkin Elmer). PNGaseF (New England BioLabs), an *N*-glycosidase, was used to remove *N*-glycans on IGF-1Rα.

### Receptor dimerization assay

SH-SY5Y (Mock and GALNT2 transfectants) and SK-N-DZ (si-control and si-GALNT2 knockdown) cells were seeded into 6-well plates. The cells were placed on ice for 15 minutes and then treated with IGF-1 (50 ng/ml) at 37ºC for 5 minutes. The transfectants were washed twice with PBS and then incubated with BS^3^ crosslinker (Sulfo-DSS) (Thermo Scientific Pierce) in PBS at 37ºC for 15 minutes. After two PBS washes, cells were lysed in NP-40 lysis buffer (Tris 20 nmol/L, PH8.0, NaCl 137mmol/L, 1% NP-40, 10% glycerol, Na_3_VO_4_ 2 mmol/L, β-glycerophosphate 2 mmol/L, PMSF 2mmol/L and 1% protease inhibitor cocktail from Sigma-Aldrich). β-actin was used as an internal control.

### Statistical analyses

Data are presented as the mean ± SD. Statistical analyses were performed using SPSS 10.0 for Windows software (SPSS Inc.). The Student *t* test was used to compare differences between two experimental groups. The Pearson χ2 test was used to assess associations between pairs of categorical variables. The Kaplan-Meier method was performed to estimate survival probabilities in various subgroups and significant differences between groups were established using log-rank tests. Each factor possibly affecting patient survival was further analyzed using univariate and multivariate Cox proportional hazards model analysis. All statistical tests were 2-sided, and *P* < 0.05 was considered statistically significant.

## SUPPLEMENTARY MATERIAL AND FIGURES


