# Supplementary material for: GALNT2 suppresses malignant phenotypes through IGF-1 receptor and predicts favorable prognosis in neuroblastoma
Source: Oncotarget. 2014 Oct 24;5(23):12247–59. doi: 10.18632/oncotarget.2627 (PMC4322969; doi:10.18632/oncotarget.2627)
Supplement: Supplementary file 1 [file oncotarget-05-12247-s001.pdf]

GALNT2 suppresses malignant phenotypes through IGF-1 receptor and predicts favorable prognosis in neuroblastoma

Supplementary Material

Transfection

SH-SY5Y and SK-N-AS cells were cultured on 6-well plates at 90% confluence. Different concentrations (1 μg, 2 μg, and 4 μg) of GALNT2/pcDNA3.1B plasmid were prepared and incubated with different volumes (2 μl, 5 μl, and 10 μl) of Lipofetamine 2000 (Invitrogen, Life Technologies) for low-, medium-, and high-expression with serum-free DMEM, respectively. After 30 minutes of incubation, transfection mixtures were applied to each well for 16 hours. The transfected cells were selected with 400 μg/ml G418 for two weeks.

Western blot analysis and VVA-lectin pull-down assay

GALNT2 expression level was detected by the rabbit anti-GALNT2 polyclonal antibody (Sigma-Aldrich). For detection of IGF-1R, rabbit anti-IGF-1Rα polyclonal antibody (Santa Cruz Biotechnology, Inc.) was used. In lectin pull-down assays, 300 μg protein lysates were prepared and pretreated with neuraminidase (Sigma-Aldrich) at 37°C for 1 hour and incubated with VVA-lectin beads (Vector Laboratories) on shaker at 4°C for 16 hours. In the binding specificity of VVA-lectin experiment, 100 μM GalNAc (Sigma-Aldrich) was preincubated with VVA-lectin beads at 4°C for 6

hours. After two PBS washes, 300 μg protein lysates were applied and incubated on shaker at 4ºC for 16 hours. The pulled down proteins were subjected to Western blot analysis with the rabbit anti-IGF-1Rα polyclonal antibody (Santa Cruz Biotechnology, Inc.).

Analysis of glycophenotypes in NB cells by flow cytometry

SH-SY5Y, SK-N-AS, and SK-N-DZ (1×10^5) cells were trypsinized and resuspended in 100 μl PBS. Neuraminidase (Sigma-Aldrich) was added and incubated at 37ºC for 30 minutes to remove sialic acid structures. After two PBS washes, fluorescein VVA-, PNA-, MAL- and SNA-lectin at 1:100 were added and incubated at 4ºC for 30 minutes. After two PBS washes, the fluorescence intensity of 1×10^4 cells per sample was analyzed by flow cytometry (FACS Calibur; BD Pharmingen). Samples without fluorescein lectin served as negative controls for each cell.

MTT assay

Cells were seeded in 96-well plates, and each well contained 2×10^3 cells in 100 μl DMEM with 10% FBS. AG1024 (Calbiochem) dissolved with DMSO (Sigma) as IGF-1 inhibitor was added to the final concentration at 5 μM and equal volume of DMSO was added as control. 10 μl of 5 mg/ml

3-(4,5-dimethyl-2-thiazolyl)-2,5-diphenyl-2H-tetrazolium bromide solution (MTT; Sigma) was added to each well for the indicated times. Cells were incubated with MTT solution at 37ºC for 4 hours. To dissolve the MTT formazan crystals, 100 μl 10% SDS in 0.01N HCl was added. The colorimetrical intensity was measured at the dual wavelengths of 550 and 630 nm using a spectrophotometer.

Transwell migration and Matrigel invasion assay

Transwell migration assays were performed using 6.5-mm polycarbonate transwell filters with 8-μm pores (Corning). SK-N-DZ transfectant cells (5×10^4) in 300 μl serum-free DMEM were seeded to the upper surface of the transwell chamber, whereas 700 μl in serum-free DMEM containing 50 ng/ml IGF-1 as chemoattractant was loaded into the lower chamber of 24-well plates. BioCoat Matrigel invasion chambers (BD Pharmingen) were used for the cell invasion assay. Cells were allowed to migrate toward the transwell chamber or invade the Matrigel for 24 hours. For IGF-1 inhibitor experiment, AG1024 (Calbiochem) was added to upper chamber to the final concentration at 5 μM and the same volume of DMSO (Sigma) was added to the control group. The migrated and invaded cells were fixed with 100% methanol and stained with 0.5% (w/v) crystal violet (Sigma). The numbers of migrated and

invaded cells per field were counted in at least three independent experiments (mean ± SD).

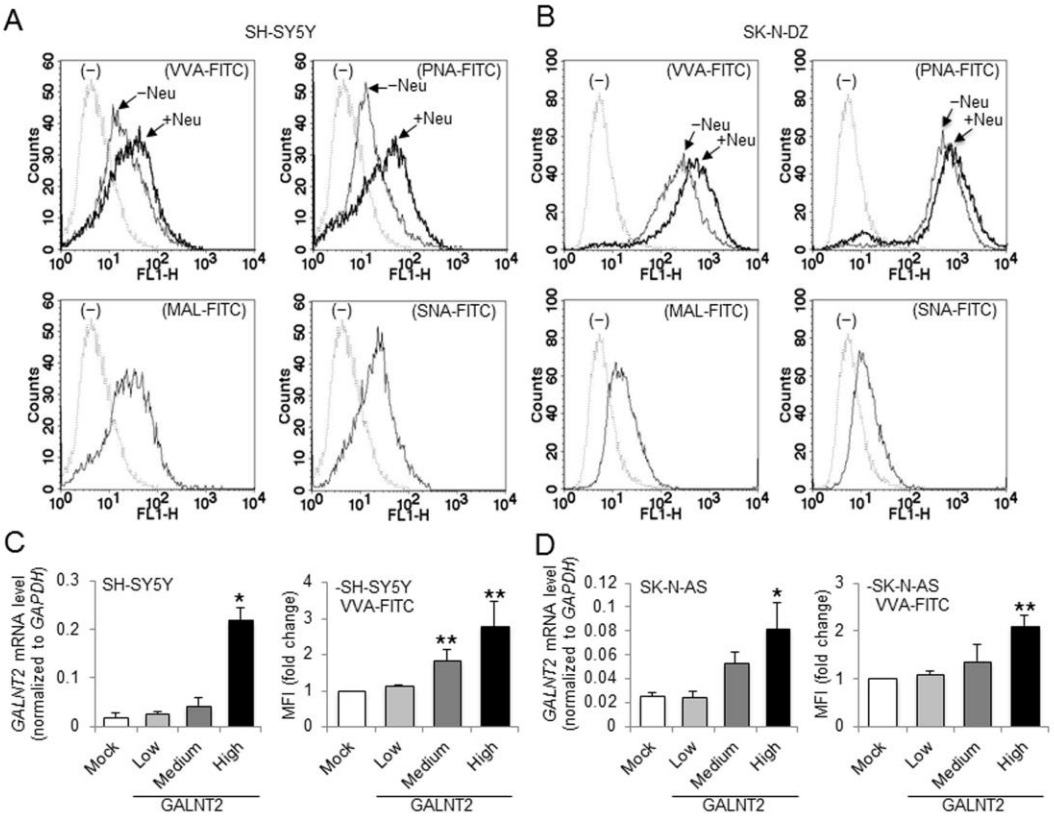

Supplementary Figure S1: Cell surface expression patterns of Tn-, T-, sTn, and sT-antigens in NB cells: Neuraminidase was used to removal terminal sialic acids on glycans. VVA- and PNA-lectins were used to recognize Tn- and T-antigens, respectively. MAL- and SNA-lectins were used to recognize α(2,3)- and α(2,6)-linked sialic acid structures, respectively. (A–B) Flow cytometry in upper panels revealed that parental SH-SY5Y and SK-N-DZ cells expressed Tn-, T-, sTn- and sT-antigens on the cell surface using VVA-FITC and PNA-FITC. Lower panels showed that α(2,3)- and α(2,6)-linked sialic acid structures were expressed in parental SH-SY5Y

and SK-N-DZ cells using MAL-FITC and SNA-FITC, respectively. (C–D) SH-SY5Y and SK-N-AS cells were transfected with 4 µg of empty vectors (Mock) and 1 µg, 2 µg, and 4 µg of *GALNT2*/pcDNA3.1B plasmids for establishing three clones with low, medium, and high expression of GALNT2, respectively. Transfected cells were selected with 400 µg/mL of G418 for 14 days and then pooled for further studies. GALNT2 mRNA levels were analyzed by real-time PCR and normalized to *GAPDH* (Supplementary Figure S1C and S1D, left). Mean fluorescence intensities (MFI) detected by flow cytometry using VVA-FITC after pretreatment with neuraminidase revealed that clones expressing a higher level of GALNT2 showed a higher amount of Tn antigen on the surface of NB cells (Supplementary Figure S1C and S1D, right). Data are presented as mean ± SD from three independent experiments. Error bar = SD. *$P < 0.05$, **$P < 0.01$

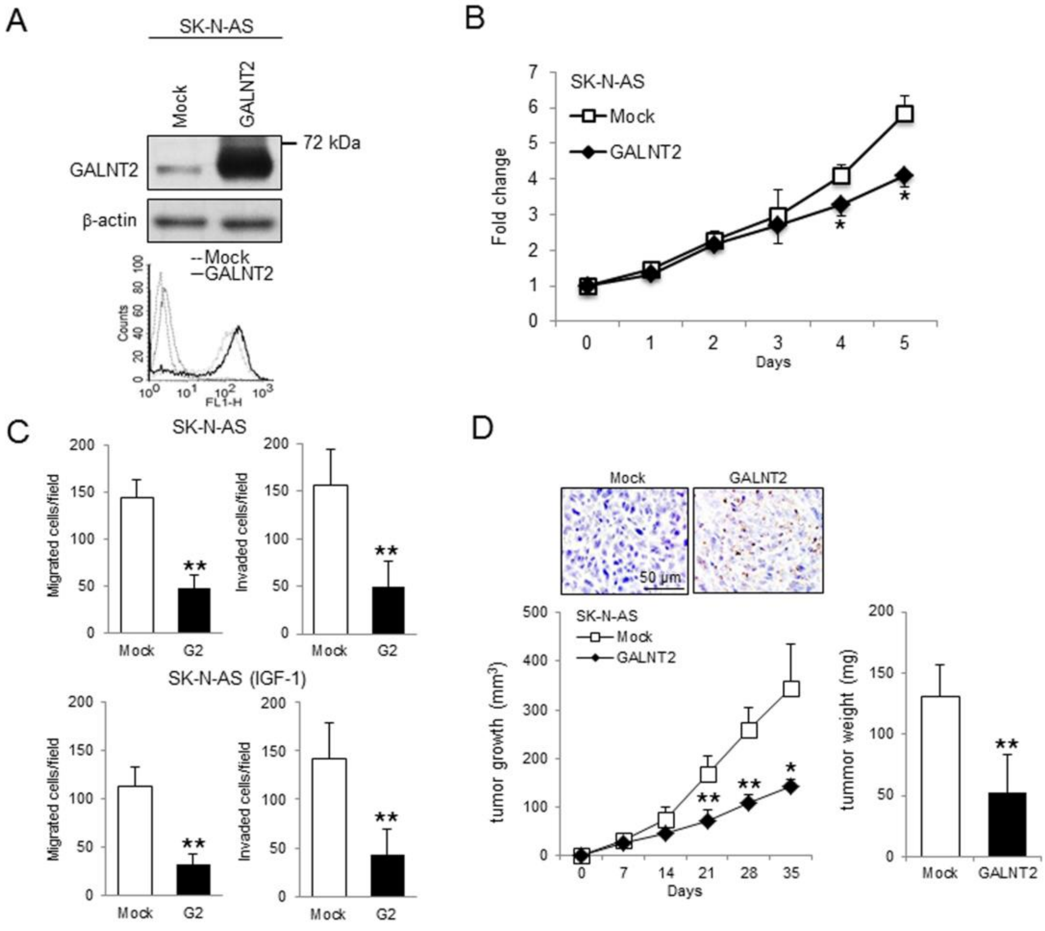

Supplementary Figure S2: Effects of GALNT2 on malignant phenotypes in SK-N-AS cells. (A) Overexpression of GALNT2 in SK-N-AS cells was confirmed using Western blotting. β-actin served as the internal control (upper panel). Flow cytometry using VVA-FITC revealed that GALNT2 overexpression increased the amount of GalNAc on the surface of SK-N-AS cells compared with controls (lower panel). (B) MTT assay revealed that GALNT2 overexpression significantly suppressed SK-N-AS cell growth. Cells were cultured in DMEM containing 10% FBS, and MTT reagent was added to cells at the indicated times. The results were standardized by setting the value at day 0 to 1.0. Data are presented as mean ± SD

from three independent experiments. Error bar = SD. *$P < 0.05$. (C) GALNT2 overexpression in SK-N-AS cells (G2) inhibited FBS-induced (upper panel) and IGF-1-induced (lower panel) migration and invasion compared with controls (Mock). Cells were seeded in serum-free DMEM and the chemoattractant in the lower chamber was 10% FBS or 50 ng/mL IGF-1. Data represent the mean ± SD for three independent experiments. **$P < 0.01$. (D) GALNT2 suppressed tumor growth in mice. SK-N-AS transfectants were subcutaneously injected to mice. After implantation, tumor sizes were measured twice a week. At day 35, tumors were excised, weighed, and subjected to immunohistochemical staining using the anti-GALNT2 antibody (color images). Scale bar = 50 µm. Original magnification, 400×. Data represent the mean ± SD; $n = 4$ for each group. *$P < 0.05$, **$P < 0.01$.

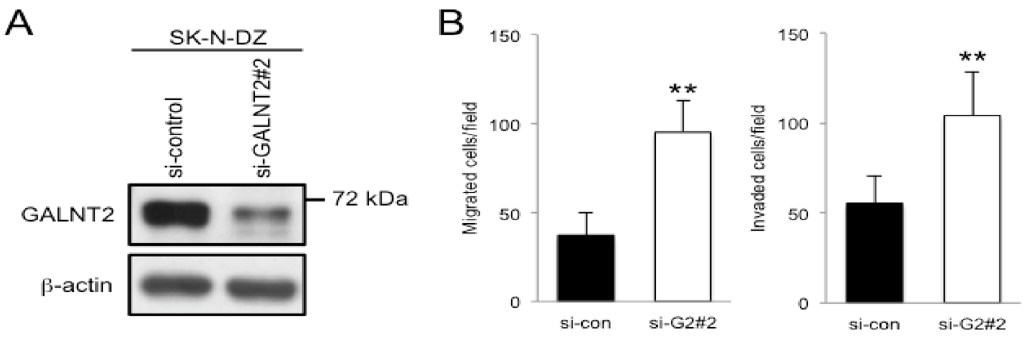

Supplementary Figure S3: Effects of GALNT2 knockdown on malignant phenotypes in SK-N-DZ cells. (A) GALNT2 knockdown in SK-N-DZ cells was confirmed using Western blotting. β-actin served as the internal control. (B–C) Cells were transfected with control siRNA (si-con) or GALNT2 siRNA (si-G2#2). GALNT2 knockdown enhanced migration (B) and invasion (C) in SK-N-DZ cells. Migration and invasion were analyzed by transwell migration and Matrigel invasion assays, respectively. Cells were seeded in serum-free DMEM and the chemoattractant in the lower chamber was 10% FBS. Data are presented as mean ± SD from three independent experiments. Error bar = SD. $^{**}P < 0.01$.

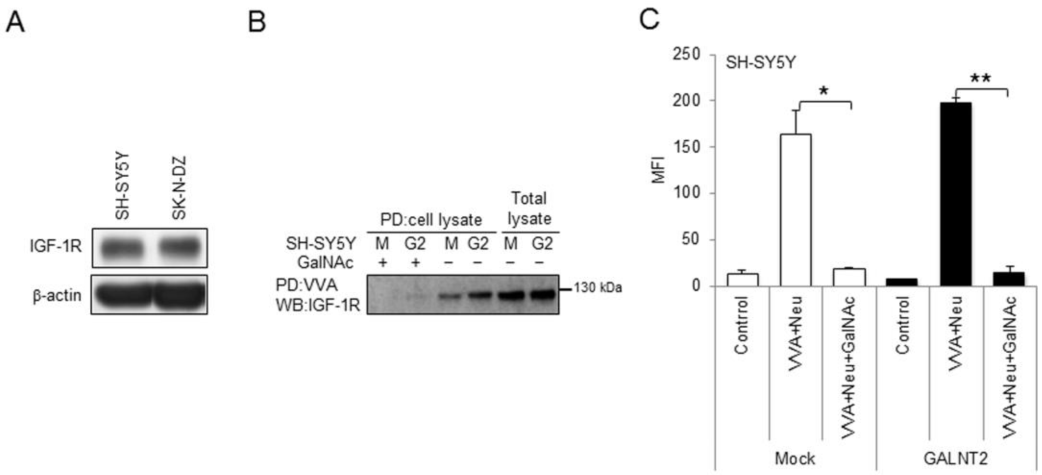

Supplementary Figure S4: VVA-lectin is specific for the Tn structure. (A) The expression levels of endogenous IGF-1R in SH-SY5Y and SK-N-DZ cells analyzed by Western blotting. (B–C) GalNAc (100 μM) was used as a competitor for VVA-lectin pull-down assays and flow cytometry. GalNAc effectively blocked in VVA binding to Tn-antigen structures on IGF-1R in SH-SY5Y cells. Data represent the mean ± SD from three independent experiments. Error bar = SD. *$P < 0.05$, **$P < 0.01$.

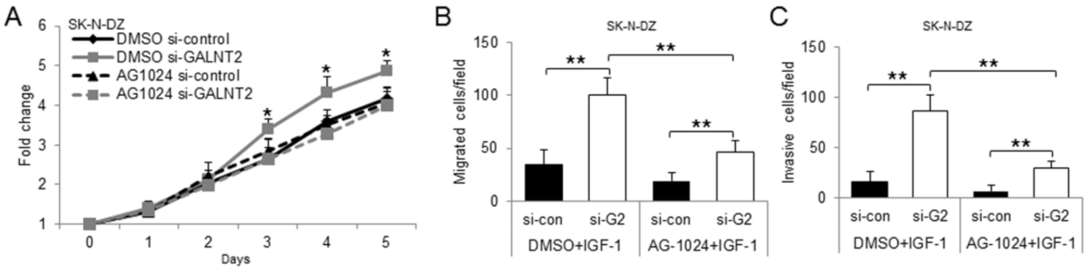

Supplementary Figure S5: IGF-1 inhibitor AG1024 suppresses malignant phenotypes in GALNT2-knockdown SK-N-DZ cells. (A) MTT assays showed that AG1024 suppressed cell growth in SK-N-DZ cells. (B–C) AG1024 significantly inhibited IGF-1-triggered cell migration and invasion in GALNT2-knockdown SK-N-DZ cells. AG1024 (5 μM) was added to the complete medium for MTT assays and serum-free DMEM in the upper chamber of Transwell for migration and invasion assays. Data represent the mean ± SD from three independent experiments. Error bar = SD. *P < 0.05, **P < 0.01.
